# Ambiguity of non-systematic chemical identifiers within and between small-molecule databases

**DOI:** 10.1186/s13321-015-0102-6

**Published:** 2015-11-16

**Authors:** Saber A. Akhondi, Sorel Muresan, Antony J. Williams, Jan A. Kors

**Affiliations:** Department of Medical Informatics, Erasmus University Medical Centre, P.O. Box 2040, 3000 CA Rotterdam, The Netherlands; Food Control Department, Banat University of Agricultural Sciences and Veterinary Medicine, Calea Aradului 119, 300645 Timisoara, Romania; ChemConnector Inc., 904 Tamaras Circle, Wake Forest, NC 27587 USA

**Keywords:** Molecular structure, Chemical databases, Non-systematic chemical identifiers, Chemical name ambiguity, Quality control

## Abstract

**Background:**

A wide range of chemical compound databases are currently available for pharmaceutical research. To retrieve compound information, including structures, researchers can query these chemical databases using non-systematic identifiers. These are source-dependent identifiers (e.g., brand names, generic names), which are usually assigned to the compound at the point of registration. The correctness of non-systematic identifiers (i.e., whether an identifier matches the associated structure) can only be assessed manually, which is cumbersome, but it is possible to automatically check their ambiguity (i.e., whether an identifier matches more than one structure). In this study we have quantified the ambiguity of non-systematic identifiers within and between eight widely used chemical databases. We also studied the effect of chemical structure standardization on reducing the ambiguity of non-systematic identifiers.

**Results:**

The ambiguity of non-systematic identifiers within databases varied from 0.1 to 15.2 % (median 2.5 %). Standardization reduced the ambiguity only to a small extent for most databases. A wide range of ambiguity existed for non-systematic identifiers that are shared between databases (17.7–60.2 %, median of 40.3 %). Removing stereochemistry information provided the largest reduction in ambiguity across databases (median reduction 13.7 percentage points).

**Conclusions:**

Ambiguity of non-systematic identifiers within chemical databases is generally low, but ambiguity of non-systematic identifiers that are shared between databases, is high. Chemical structure standardization reduces the ambiguity to a limited extent. Our findings can help to improve database integration, curation, and maintenance.

**Electronic supplementary material:**

The online version of this article (doi:10.1186/s13321-015-0102-6) contains supplementary material, which is available to authorized users.

## Background

A wide range of chemical compound databases are currently available for pharmaceutical research [1]. They provide a variety of chemical information [2], most importantly compound structures, which can be used for different purposes, such as chemical predictive modelling [3] or quantitative structure–activity relationships modelling [4]. To retrieve information about a compound, researchers can query these chemical databases using one of many available compound identifiers. Information retrieval based on automatic extraction of chemical identifiers from scientific literature or patents, is becoming increasingly important as the large amount of such unstructured texts makes manual extraction and analysis cumbersome [5–7]. Text mining methods that extract compound-target or drug-disease relationships from text, can provide valuable new insights [8] or support database curation [9, 10]. The correctness of the chemical identifiers that link to the chemical structures in the databases can greatly affect the results of cheminformatics analyses [11, 12].

Chemical identifiers fall into two main classes. The first class consists of systematic identifiers, which are algorithmically defined based on the chemical structure of the compound [13]. Among the systematic identifiers are IUPAC names [14], SMILES [15], and International Chemical Identifiers (InChIs) [16, 17]. We have previously investigated the correctness or consistency of systematic identifiers (i.e., whether an identifier matches the associated structure) within and across small-molecule databases, and found many inconsistencies [13]. We also checked whether the inconsistencies could be reduced by different chemical structure standardizations (e.g., removal of fragments, or ignoring isotopes), but this was only the case to a limited extent [13].

The second class of chemical identifiers consists of non-systematic identifiers. These are source-dependent identifiers which are usually assigned to the compound at the point of registration in a chemical database [13]. Brand names, generic names, research codes, chemical abstracts service (CAS) registry numbers, and database identifiers are examples of such non-systematic identifiers. Since there is no algorithmic relationship between non-systematic identifiers and structures, the correctness of these identifiers can only be assessed manually, which has proven cumbersome [1]. However, it is possible to automatically check the ambiguity of non-systematic identifiers (i.e., whether an identifier matches more than one structure). The extent of this ambiguity problem is unknown and not yet quantified.

Here, we investigate the ambiguity of non-systematic identifiers within and between small-molecular databases, before and after chemical structure standardisation.

## Methods

### Databases

We selected eight well-known chemical databases covering a wide range of bioactive compounds: Chemical Entities of Biological Interest (ChEBI) [18], ChEMBL [19], ChemSpider [20], DrugBank [21], the Human Metabolome Database (HMDB) [9, 22], the NCGC Pharmaceutical Collection (NPC) [23], PubChem [24], and the Therapeutic Target Database (TTD) [25, 26]. We focused on compound records that had associated chemical structures in the form of MOL files [27]. For each record, we extracted the structure file and gathered all chemical identifiers (available from possibly different record fields), except for identifiers explicitly tagged as IUPAC names, SMILES, or InChIs. For example, identifiers for the antibiotic “ampicillin” included “ampicilina”, “ampicillin acid”, “AMP”, “AP”, “ABPC”, “ay-6108”, “DB00415”, “penbritin”, “totacillin”, “PEN A/N”, “Prestwick3_000114”, “Ampi-bol”, “Aminobenzylpenicillin” and, “brl 1341”. Note that extracted identifiers may include database identifiers (such as “DB00415”) that appear in the name fields of the chemical records. Typically, for a given chemical database, database identifiers in its name fields come from other databases, and local database identifiers are only used as record identifiers (and not extracted). All data were downloaded in February 2013. The identifiers extracted from all databases, except ChemSpider which is a commercial database, are made available through http://www.biosemantics.org. In the following, we briefly describe the databases, indicating the version that was used (if versioning was available) and the fields from which identifiers were extracted.

*ChEBI* is a database of molecular entities, focusing on small chemical compounds [18]. ChEBI provides an ontological classification with parent and child relationships. We extracted data for all three-star (i.e., manually annotated) compounds from ChEBI SD files. This included synonyms, ChEBI names, brand names, and International Non-proprietary Names (INN).

*ChEMBL* is a large-scale bioactivity database containing information for drug-like bioactive compounds [19]. In addition to literature-derived data ChEMBL also contains Food and Drug Administration (FDA) approved drugs. The data available through ChEMBL have been manually extracted and standardized [19]. We used a local installation of ChEMBL version 14. Extracted fields include preferred name, synonyms, FDA alternative names, trade names, INN, United States Adopted Names (USAN), and United States Pharmacopoeia names (USP).

*ChemSpider* is a chemical database containing information of compounds gathered from over 500 different data sources [20]. ChemSpider structures and their corresponding identifiers were made available from the Royal Society of Chemistry (RSC)[28]. We focused on compounds that have structure–activity relationships or other biological annotations. Similar selection criteria as defined by Muresan et al. [29] were provided to the ChemSpider team to extract the ChemSpider data. Subsets of chemicals such as “make on demand” chemicals from screening library vendors without names other than computationally generated systematic names were excluded, as were the datasets that have been deprecated from ChemSpider during curation. We also considered a subset of the ChemSpider data that only contained information that was validated with the use of crowdsourcing, including curation work performed by members of the ChemSpider technical support team (ChemSpider-V) [20, 30]. For each compound, we were provided with all preferred terms and synonyms.

*DrugBank* provides information regarding drugs, including chemical, pharmacological and pharmaceutical drugs and their targets [21]. DrugBank data are curated by a curation team based on primary literature sources. During production and maintenance all synonyms and brand names within DrugBank are extensively reviewed and only the most common synonyms are kept [31]. We used DrugBank version 3.0, and extracted generic names, synonyms, CAS numbers, and brand names from the DrugBank SD files and DrugCards.

*HMDB* contains small-molecule metabolites found in the human body. The database links chemical, clinical, molecular-biology, and biochemistry data. HMDB is both automatically and manually curated [9, 22]. We used HMDB version 3. All generic names, CAS numbers, and synonyms were extracted from HMDB SD files and MetaboCards.

*NPC* provides clinically-approved drugs from USA, Europe, Canada, and Japan for high-throughput screening [23]. In addition NPC provides chemical-related information gathered from different sources, such as the KEGG database. Using NPC browser 1.1.0, we extracted preferred names and synonyms.

*PubChem* is a database that provides information on the biological activities of small molecules [24]. PubChem consists of three different databases: a compound database (with currently about 61 million entries), a substance database (about 157 million entries), and a bioassay database (more than 1 million entries). The compound database was used to extract structures for a subset of compounds that had structure–activity relationships or other biological annotations. This subset of compounds was introduced by Muresan et al. [29] and is the same subset of PubChem compounds that we used in our previous study on the consistency of systematic identifiers [13]. The PubChem compound database does not contain non-systematic identifiers. This information is available through the PubChem substance database. The relations between PubChem substance identifiers (SIDs) and compound identifiers (CIDs), which have been created by PubChem through in-house chemical structure standardization [24], are specified in the “PubChem_CID_associations” tag available in the downloadable SD files [32]. We used the relations between SIDs and CIDs to extract the non-systematic identifiers (synonyms and identifiers) from the substance database and assign them to the corresponding compounds [24].

*TTD* provides therapeutic protein and nucleic acid targets and drug information including targeted disease and pathway [25, 26]. We used TTD version 4.3.02. All synonyms, trade names, and drug names were extracted.

### Filtering

The fields with non-systematic identifiers that were extracted from the databases may also contain systematic identifiers (e.g., a field with synonyms may not distinguish between the two types of identifiers). Systematic identifiers were automatically filtered out from the extracted identifiers with the use of two name-to-structure converters, ChemAxon’s MolConverter [33] and the open source tool OPSIN (Open Parser for Systematic IUPAC Nomenclature) [34]. Both tools are freely available for academic research. We used two different name converters since the algorithms that they implement to recognize systematic identifiers may differ slightly (mostly when considering IUPAC names). Each extracted identifier was fed into the converters and only considered non-systematic if neither tool recognized it as systematic. For example, the term “(2*S*, 5*R*, 6*R*)-6-{[(2*R*)-2-amino-2-phenylacetyl]amino}-3,3-dimethyl-7-oxo-4-thia-1-azabicyclo[3.2.0]heptane-2-carboxylic acid” was not labelled as a IUPAC name in DrugBank “DB00415” but it was filtered out through this step.

### Ambiguity within and across databases

A non-systematic identifier was considered ambiguous within a database if it appeared in multiple records in the database, i.e., if multiple structures were provided for the same identifier. Ambiguity was measured as the percentage of unique identifiers within a database that are ambiguous.

An identifier was considered ambiguous across two databases if the structures (as defined by their MOL files) of the compounds associated with the identifier in the two databases were different. If an identifier was ambiguous in one or both of the databases (i.e., the identifier was associated with multiple compounds within the database(s)), the identifier was also considered ambiguous across databases. Ambiguity was calculated as the percentage of unique shared identifiers between databases that are ambiguous.

To compare two MOL files, we used the same approach as in our previous study [13]. Briefly, each MOL file was converted into a Standard InChI with ChemAxon’s MolConverter [33], providing a unique textual representation of the MOL file. The two InChI strings were then compared to determine whether the corresponding structures were the same. No comparison was made if an InChI could not be generated.

### Standardization

In the process of creating MOL files for compounds, databases can apply different sensitivity settings [2]. These settings pertain to including or ignoring fragments, isotopic labels, charges, canonical tautomers, or stereochemical information. Different sensitivity settings can result in different Standard InChI strings for the same compound, and thus are a potential source of ambiguity. Standardization of the MOL files can help to reduce such ambiguities.

The Computer-Aided Drug Design group of the National Cancer Institute defined a set of rules called FICTS to standardize the structural representation of compounds [2, 35]. FICTS rules correspond to five standardisation levels that affect structural information. The rules remove small fragments (F), disregard isotopes (I) and charges (C), generate canonical tautomers (T), or ignore stereochemical information (S). Any combination of the five rules can be applied and is expressed by converting the corresponding upper-case letter of the term “FICTS” into a “u” (for “un-sensitive”). ChemAxon’s Standardizer [36] was used to execute these standardization rules.

## Results

### Databases

For each database, Table 1 shows the number of compounds with at least one non-systematic identifier, and the total number of non-systematic identifiers (not unique). The databases vary greatly in size and in the average number of non-systematic identifiers per compound, ranging from 1.3 for ChemSpider-V and ChEMBL to 35.4 for TTD. The large average for TTD can be attributed to the presence of a large number of database identifiers for many of the compounds.

**Table 1:** Number of compounds and non-systematic identifiers in different chemical databases

Database	Compounds	Identifiers	Identifiers/compound
PubChem	4,232,875	15,211,133	3.6
ChemSpider	6,646,902	10,063,709	1.5
ChemSpider-V	654,052	850,601	1.3
HMDB	37,761	308,733	8.2
NPC	14,814	131,290	8.9
TTD	2977	105,407	35.4
ChEBI	15,633	41,956	2.7
ChEMBL	21,398	28,011	1.3
DrugBank	3769	26,780	7.1

### Ambiguity of non-systematic identifiers within databases

Table 2 shows the ambiguity of non-systematic identifiers and the average number of compounds per ambiguous identifier within the databases. HMDB has 15.2 % ambiguity, much larger than for any of the other databases. On average, an ambiguous identifier in HMDB is associated with 6.1 compounds, but the distribution is highly skewed. For example, the two most ambiguous identifiers in HMDB, “Triglyceride” and “Triacylglycerol”, are each associated with about 14,000 compounds. Moreover, HMDB contains 176 non-systematic identifiers with more than 100 structures (100 being an arbitrary number chosen for the purpose of comparison). The only other databases that contain identifiers that are associated with more than 100 structures, are ChemSpider (39 identifiers) and PubChem (16 identifiers). Some of these identifiers are unspecific, e.g., “ester” is linked to 228 structures in ChemSpider.

**Table 2:** Ambiguity of non-systematic identifiers and the average number of compounds per ambiguous identifier, within databases

Database	Unique identifiers	Ambiguous identifiers	Ambiguity (%)	Compounds/ambiguous identifier
HMDB	173,455	26,430	15.2	6.1
TTD	100,570	4607	4.6	2.1
ChEMBL	26,910	1050	3.9	2.1
NPC	112,717	3455	3.1	2.1
ChemSpider	9,691,277	245,541	2.5	2.5
ChEBI	41,023	827	2.0	2.1
PubChem	14,937,728	201,621	1.3	2.4
ChemSpider-V	842,128	5401	0.6	2.3
DrugBank	26,759	20	0.1	2.1

TTD is the database with the second-largest ambiguity (4.6 %), but none of the ambiguous identifiers in TTD are associated with more than three compounds. This is also reflected in the low average number of compounds per ambiguous identifier (2.1), close to the minimum of 2 that would be reached if all ambiguous identifiers were associated with exactly two compounds. The ambiguity of ChemSpider-V (0.6 %) is much lower than the ambiguity of ChemSpider (2.5 %), suggesting a positive effect of curation. However, when we recalculated the ambiguity of the ChemSpider-V records prior to curation, we found an ambiguity of 0.7 %. Therefore, the curation effort only slightly reduced ambiguity within ChemSpider-V, possibly because it focused more on establishing the correctness of compound structures. DrugBank has the lowest ambiguity of non-systematic identifiers (0.1 %).

### Ambiguity of non-systematic identifiers between databases

Table 3 presents for each pair of databases the number of unique non-systematic identifiers that are shared between the databases. The first figure in the parentheses indicates the ambiguity of these shared identifiers, i.e., the percentage of shared identifiers for which the corresponding structures in the two databases are different. For example, the identifier “floxuridine” occurs in ChEBI and in ChEBML, but the corresponding structures in these two databases do not match, and thus the identifier is ambiguous. The second figure in the parentheses shows the percentage of the shared identifiers that are ambiguous within one or both of the databases, and thus are ambiguous across databases by definition. For example, “ofloxacin” is shared between ChEMBL and HMDB, but is also ambiguous within HMDB because it is associated with two different structures (in records HMDB01929 and HMDB15296). Therefore, the identifier is considered ambiguous, even though one of the structures in HMDB (HMDB15296) matches the one in ChEMBL.

**Table 3:** Number of shared non-systematic identifiers between databases, ambiguity of the shared identifiers (first figure in parentheses, in italics), and the percentage of shared identifiers that are ambiguous within at least one of the databases (second figure in parentheses)

Database	ChEBI	ChEMBL	ChemSpider	ChemSpider-V	DrugBank	HMDB	NPC	PubChem
ChEMBL	1886 (*39.5*/18.2)							
ChemSpider	28,281 (*30.9*/24.1)	23,584 (*29.9*/22.3)						
ChemSpider-V	5081 (*39.9*/9.8)	4303 (*43.6*/16.6)						
DrugBank	2981 (*28.7*/3.4)	4108 (*39.6*/14.7)	19,222 (*50.7*/32.0)	6985 (*45.2*/6.7)				
HMDB	4529 (*49.6*/10.6)	2325 (*48.4*/17.7)	27,608 (*57.3*/29.5)	11,774 (*43.9*/8.8)	5515 (*30.7*/5.2)			
NPC	5516 (*40.7*/6.1)	6858 (*46.4*/15.1)	62,527 (*60.2*/26.8)	18,709 (*48.6*/7.4)	22,377 (*21.9*/2.0)	6815 (*44.4*/7.4)		
PubChem	24,331 (*36.9*/26.1)	25,607 (*33.1*/28.9)	2,275,338 (*17.7*/8.5)	99,334 (*41.6*/19.2)	24,929 (*46.8*/39.4)	35,905 (*43.3*/28.3)	68,280 (*49.8*/29.6)	
TTD	4854 (*27.7*/7.6)	5019 (*36.9*/16.9)	50,182 (*32.3*/18.4)	8305 (*40.3*/10.4)	17,232 (*18.2*/6.8)	6256 (*43.0*/11.2)	23,669 (*22.4*/6.9)	98,853 (*25.4*/23.0)

Ambiguity between two databases varies widely, from 17.7 % (for PubChem and ChemSpider) to 60.2 % (for NPC and ChemSpider). Overall, the lowest ambiguity values between a given database and the other databases are seen for TTD (median ambiguity over all databases 30.0 %), while highest values occur for NPC (median 45.4 %), and HMDB (median 44.2 %).

The percentage of shared identifiers that are ambiguous within either or both of the databases (i.e., are ambiguous across databases by definition) also varies greatly. For instance, 39.4 % of the shared identifiers between DrugBank and PubChem are also ambiguous within the databases, largely accounting for the overall ambiguity of 46.8 %. (This means that only 7.4 % of the shared identifiers are ambiguous across but not within the databases.) Similar values are seen for ChEMBL and PubChem (33.1 % overall ambiguity and 28.9 % ambiguity due to identifiers that are ambiguous within the databases) and PubChem and TTD (25.4 and 23.0 %, respectively). On the other hand, for DrugBank and NPC only 2.0 % ambiguity is due to ambiguous identifiers within the databases (overall ambiguity 21.9 %), and for DrugBank and ChEBI only 3.4 % (overall 28.7 %).

### Effect of standardisation

Table 4 shows the effect of different types of standardization on reducing the ambiguity of non-systematic identifiers within databases. For most databases, standardization has little effect on ambiguity (median change for each setting less than 0.5 percentage point). The largest changes are seen for TTD and ChEMBL, in particular for removing fragments (uICTS). Overall, removing fragments and disregarding stereochemistry (FICTu) gives the largest changes, while disregarding isotopes (FuCTS) has the lowest effect. Notably, standardization does not affect HMDB, the most ambiguous database.

**Table 4:** Effect of standardization on the ambiguity of non-systematic identifiers (in  %) within databases

Database	FICTS	uICTS	FuCTS	FIuTS	FICuS	FICTu
HMDB	15.2	15.2	15.2	15.2	15.2	15.2
TTD	4.6	1.8	2.1	2.0	2.1	2.1
ChEMBL	3.9	2.0	3.8	3.9	3.9	3.4
NPC	3.1	2.7	2.7	2.7	2.7	2.7
ChemSpider	2.5	2.3	2.5	2.5	2.2	1.9
ChEBI	2.0	1.8	1.9	1.4	1.8	1.6
PubChem	1.4	1.2	1.3	1.3	0.6	0.6
ChemSpider-V	0.6	0.6	0.6	0.6	0.6	0.3
DrugBank	0.1	0.1	0.1	0.1	0.1	0.1

We also computed the effect of different standardization settings on the ambiguity of non-systematic identifiers across databases. Table 5 shows the results for removing fragments (uICTS) and disregarding stereochemistry (FICTu), which gave the largest reductions in ambiguity. Results for the other standardization settings (FuCTS, FIuTS, and FICuS) are available as Additional file 1.

**Table 5:** Effect of standardization on the ambiguity of non-systematic identifiers (in  %) across databases

Database	Standardization	ChEBI	ChEMBL	ChemSpider	ChemSpider-V	DrugBank	HMDB	NPC	PubChem
ChEMBL	FICTS	39.5							
	uICTS	22.0							
	FICTu	32.6							
ChemSpider	FICTS	30.9	29.9						
	uICTS	28.4	25.0						
	FICTu	19.5	17.8						
ChemSpider-V	FICTS	39.9	43.6						
	uICTS	36.5	34.1						
	FICTu	26.1	27.3						
DrugBank	FICTS	28.7	39.6	50.7	45.2				
	uICTS	15.5	22.6	41.4	37.2				
	FICTu	23.3	32.6	35.9	33.4				
HMDB	FICTS	49.6	48.4	57.3	43.9	30.7			
	uICTS	47.4	36.1	54.4	42.4	30.4			
	FICTu	32.3	33.0	34.4	23.3	16.1			
NPC	FICTS	40.7	46.4	60.2	48.6	21.9	44.4		
	uICTS	31.2	31.1	45.9	37.3	21.3	43.5		
	FICTu	26.8	36.2	45.1	31.6	13.5	21.2		
PubChem	FICTS	36.9	33.1	17.7	41.6	46.8	43.3	49.8	
	uICTS	32.9	25.2	16.1	37.1	37.6	40.9	38.4	
	FICTu	24.1	24.0	9.0	25.4	34.6	26.7	35.1	
TTD	FICTS	27.7	36.9	32.3	40.3	18.2	43.0	22.4	25.4
	uICTS	20.9	24.6	27.8	32.7	16.8	41.1	20.6	21.6
	FICTu	15.2	26.0	17.8	23.0	10.1	22.0	9.2	13.8

Overall, ignoring stereochemistry information gave the largest ambiguity reduction (median decrease of 13.7 percentage points), but the remaining ambiguity between databases was still considerable (median 25.4 %). The largest improvements were seen for HMDB and NPC (23.2 percentage points) and for HMDB and ChemSpider (21.9 percentage points). Removal of small fragments resulted in a median reduction in ambiguity of 4.9 percentage points. The highest reduction was obtained for ChEBI and ChEMBL (17.5 percentage points).

## Discussion

We quantified the ambiguity of non-systematic identifiers within and between eight widely used chemical databases. Our results show an ambiguity between 0.1 and 15.2 % (median 2.5 %) within databases, whereas ambiguity between databases ranged from 17.7 to 60.2 % (median 40.3 %). Standardization reduced the ambiguity to some extent. Removal of small fragments gave the largest reduction (to a median of 1.8 percentage point) in ambiguity within databases, while removing stereochemistry information provided the best improvement in reducing ambiguity (median 13.7 percentage point) across databases. Possibly, the addition of three-dimensional information to structures either by hand or through automated processes introduces an extra complexity that is responsible for the ambiguity. These results complement our findings in a previous study, where we investigated the consistency of systematic identifiers (i.e., whether a systematic identifier was consistent with the associated MOL file) and showed that this consistency varied greatly within and across databases [13].

Ambiguity of non-systematic identifiers within databases is generally low, with on average few compounds associated with an ambiguous identifier. HMDB was an outlier with 15.2 % ambiguity and an average of 6.1 compounds per ambiguous identifier. Among the most common ambiguous identifiers in HMDB are different classes of Triglyceride (TG, triacylglycerol, TAG, tracylglycerol), which is an ester derived from glycerol and three fatty acids, and Phosphatidylcholine (PC), a class of phospholipids. The IUPAC-IUB Commission on biochemical nomenclature discourages the use of “triglyceride” as the ambiguity of this identifier will result in inconsistencies [37]. Chemical compound records representing drugs, metabolites, and biochemicals of other types are usually records with a higher number of non-systematic identifiers, which might lead to a higher ambiguity. However, our results suggest that there is no clear association between number of non-systematic identifiers per compound and ambiguity within the different databases. Drugbank, for example, has a fairly large average number of identifiers per compound (7.1) but showed lowest ambiguity (0.1 %), whereas ChEMBL has a low number of identifiers per compound (1.3) but relatively high ambiguity (3.9 %).

Another reason for ambiguity is that many databases massively integrate information from other databases, but may use different standardization procedures. This can result in different compound structures that have the same, but now ambiguous, non-systematic identifiers.

The ambiguity within databases is much lower than the ambiguity across databases, which varies between 17.7 % (for PubChem and ChemSpider) and 60.2 % (ChemSpider and NPC). Factors that may affect the ambiguity between databases are the ambiguity within the separate databases, the level of (manual) database curation, and standardization procedures. The ambiguity between databases that could be attributed to identifiers that are already ambiguous within one or both of the databases, varied between 2.0 % (DrugBank and NPC) and 39.4 % (DrugBank and PubChem), but generally was considerably lower than the overall ambiguity between databases. This suggests that reducing the ambiguity within databases will only partly resolve the ambiguity across databases. It should also be noted that the ambiguity between two databases is based on the number of identifiers that the databases share, which may be much lower than the number of identifiers in either database. This explains why the ambiguity between databases for identifiers that are already ambiguous in one of the databases can be much higher than the ambiguity within databases. For example, the ambiguity between DrugBank and PubChem is 39.8 %, whereas it is only 0.1 % within DrugBank and 1.4 % within PubChem. This shows that identifiers that are ambiguous within these databases are relatively frequently shared between the databases.

Database curation does not appear to affect the level of ambiguity of shared non-systematic identifiers between databases. For instance, DrugBank and ChemSpider-V, which are both considered highly curated databases [20, 38], show that 45.2 % of the shared identifiers are ambiguous (while only 6.7 % of the ambiguity between these databases could be attributed to identifiers that were already ambiguous in the separate databases). This ambiguity ranks among the highest ambiguities between databases.

The effect of chemical structure standardization on reducing the ambiguity of non-systematic identifiers is limited. The largest reductions were seen for disregarding stereochemistry and small fragments (median ambiguity reduction of 13.7 and 4.9 percentage points, respectively), but the remaining ambiguity was still considerable. The other standardization settings that we tested hardly reduced the ambiguity.

Our study may have several implications for database curation and integration efforts. First, our findings indicate that some non-systematic identifiers are very ambiguous within databases (e.g., TG, triacylglycerol, ester). These identifiers are more likely to represent classes of chemicals than individual compounds, and may be considered for removal from the databases.

Second, our study suggests that efforts to disambiguate non-systematic identifiers should not only pay attention to ambiguity within databases, which is generally low, but also consider identifiers that are ambiguous across databases. This will reveal many ambiguous and potential problematic identifiers that will not be apparent if only single databases are considered. Our method to detect these ambiguous identifiers can provide helpful information to database curators to direct their disambiguation efforts. Crowdsourcing approaches that involve the chemical community to improve database quality [20, 29, 39], may also benefit from this information to resolve ambiguity issues. All ambiguous identifiers in this study, within and between databases, are available through http://www.biosemantics.org.

Third, our findings are relevant for database integration and maintenance. Many chemical databases are increasing their coverage by regularly integrating data from other sources [40], or existing databases are merged and made available as a new resource [41]. As mentioned in our previous study [13], integration of databases should focus on a unique representation of compounds (e.g., MOL files) as their base of integration. InChI strings derived from the MOL files have been shown to facilitate the process as they are unique and can encode multiple types of information [42], although limitations also exist [43]. Ambiguity of systematic identifiers can be reduced by regenerating them from the structures [13], but such an approach is not possible for non-systematic identifiers, which are generated at the point of registration. Our results show that there is a large ambiguity of non-systematic identifiers across databases, and suggest that the integration of these identifiers from different databases without proper manual curation can greatly increase their ambiguity. It has previously been proposed to use a voting approach to disambiguate non-systematic identifiers when integrating multiple databases, assigning the identifier to the compound to which it was most frequently associated in the databases [29], but this approach may be biased by error propagation when one database includes an erroneous identifier from another database.

Our study has several limitations. First, although we included a variety of commonly used chemical databases, the number of databases is not very large and our results may not apply to databases that were not considered. Moreover, as the content of the databases evolves over time, the ambiguity within and between databases is likely to have changed since we downloaded the data. For example, recently an effort has been made to reduce ambiguity within the ChemSpider database by using a subset of records with non-systematic identifiers that had manually been validated, and automatically removing these identifiers from any record that had not been validated. A second limitation is that we quantified the ambiguity of non-systematic identifiers within and across databases, but did not determine which of the associations between non-systematic identifiers and compounds were correct, and thus could not rank the databases on their performance in this respect. A reference set of correctly assigned non-systematic identifiers would allow such an analysis, but may be cumbersome to establish. Finally, to assess whether two structures were the same, we used one tool to convert MOL files into InChI strings. Other tools might occasionally produce different conversions, because of differences in MOL file processing, but in our previous study [13] such differences were negligible and did not significantly influence the results.

## Conclusions

Ambiguity of non-systematic identifiers within chemical databases is generally low. A much higher ambiguity was observed for non-systematic identifiers that are shared across databases. Chemical structure standardization reduces the ambiguity to a limited extent. The largest reductions are obtained when disregarding stereochemistry information or removing small fragments. The results of our study can help to improve database integration, curation and maintenance.

## Additional file

10.1186/s13321-015-0102-6 The effect of all standardization settings on reducing ambiguity of non-systematic identifiers across databases.
