# Supplementary material for: Ambiguity of non-systematic chemical identifiers within and between small-molecule databases
Source: J Cheminform. 2015 Nov 16;7:54. doi: 10.1186/s13321-015-0102-6 (PMC4646925; doi:10.1186/s13321-015-0102-6)
Supplement: Supplementary file 1 — 10.1186/s13321-015-0102-6 The effect of all standardization settings on reducing ambiguity of non-systematic identifiers across databases. [file 13321_2015_102_MOESM1_ESM.pdf]

Ambiguity of non-systematic chemical identifiers within and between small-molecule databases

Saber A. Akhondi[1§], Sorel Muresan[2], Antony J. Williams[3], Jan A. Kors[1]

Supplementary data

Appendix table 1: The effect of all standardization settings on reducing ambiguity of non-systematic identifiers across databases.

Database	FICTS	uICTS	FuCTS	FIuTS	FICuS	FICTu
ChEBI-ChEMBL	39.50	21.95	39.45	38.39	37.51	32.56
ChEBI-ChemSpider-V	39.85	36.47	39.76	36.37	36.40	26.14
ChEBI-DrugBank	28.65	15.53	28.65	27.31	26.82	23.25
ChEBI-HMDB	49.59	47.41	49.35	44.78	44.91	32.28
ChEBI-NPC	40.68	31.18	40.63	40.17	38.76	26.81
ChEBI-PubChem	36.92	32.92	35.77	34.42	34.96	24.13
ChEBI-TTD	27.69	20.87	27.69	26.89	27.18	15.18
ChEMBL- ChemSpider-V	43.64	34.12	43.57	43.55	41.85	27.33
ChEMBL- DrugBank	39.56	22.59	39.53	39.39	38.88	32.57
ChEMBL- HMDB	48.43	36.13	48.26	48.39	47.61	33.03
ChEMBL- NPC	46.41	31.07	46.34	46.40	45.55	36.16
ChEMBL- PubChem	33.07	25.20	32.69	33.01	30.91	23.96
ChEMBL- TTD	36.94	24.58	36.83	36.77	35.84	25.98
ChemSpider-V- DrugBank	45.24	37.21	45.23	45.15	40.93	33.41
ChemSpider-V- HMDB	43.92	42.37	43.91	42.56	43.00	23.34
ChemSpider-V- NPC	48.60	37.31	48.55	48.51	45.49	31.63
ChemSpider-V- PubChem	41.61	37.14	41.44	41.15	39.32	25.38
ChemSpider-V- TTD	40.28	32.71	40.21	40.07	35.06	22.95
DrugBank- HMDB	30.66	30.37	30.66	30.64	29.90	16.14
DrugBank- NPC	21.92	21.26	21.92	21.90	20.78	13.53
DrugBank- PubChem	46.78	37.58	46.62	46.60	45.43	34.63
DrugBank- TTD	18.22	16.78	18.22	18.09	17.45	10.14
HMDB- NPC	44.42	43.46	44.37	44.31	43.76	21.21
HMDB- PubChem	43.32	40.86	43.01	42.73	42.34	26.71
HMDB- TTD	43.00	41.11	43.00	42.92	41.56	22.01
NPC- PubChem	49.75	38.41	49.52	49.63	36.81	35.14
NPC- TTD	22.36	20.64	22.31	22.14	21.12	9.18
PubChem- TTD	25.38	21.60	25.28	25.15	23.87	13.77
ChemSpider- ChEBI	30.88	28.40	30.48	28.39	28.09	19.45

ChemSpider- ChEMBL	29.89	24.96	29.76	29.72	27.32	17.82
ChemSpider- DrugBank	50.67	41.44	50.60	50.20	47.87	35.94
ChemSpider- HMDB	57.28	54.36	57.12	56.25	55.71	34.35
ChemSpider- NPC	60.22	45.90	60.01	59.86	58.16	45.12
ChemSpider- PubChem	17.70	16.07	17.59	17.53	16.85	9.02
ChemSpider- TTD	32.26	27.82	32.12	31.81	29.94	17.83
